# Analysis on epidemiological and drug resistance characteristics of lymph node tuberculosis from Hunan province, China

**DOI:** 10.3389/fpubh.2024.1432065

**Published:** 2024-07-05

**Authors:** Tanwei Fang, Yu Xiang, Wenbin Li, Binbin Liu, Haican Liu, Yaning Wu, Da Xu, Machao Li, Shuliu Yang, Jixiang Li, Yanyan Yu, Xiuqin Zhao, Li-li Zhao, Kanglin Wan, Xiuqin Yuan, Yunhong Tan, Guilian Li

**Affiliations:** ^1^Hunan Institute for Tuberculosis Control & Hunan Chest Hospital, Changsha, China; ^2^National Key Laboratory of Intelligent Tracking and Forecasting for Infectious Diseases, National Institute for Communicable Disease Control and Prevention, Chinese Center for Disease Control and Prevention, Beijing, China; ^3^School of Public Health, University of South China, Hengyang, China; ^4^School of Basic Medical Sciences, Central South University, Changsha, China

**Keywords:** tuberculosis, lymph node tuberculosis, extrapulmonary tuberculosis, clinical epidemiology, drug resistance

## Abstract

**Objectives:**

To investigate the clinical epidemiological and drug resistance (DR) characteristics of lymph node tuberculosis (LNTB) in Hunan Province which locates in South-central China, and to provide scientific clues for effective prevention and treatment of LNTB.

**Methods:**

We retrospectively collected LNTB patients with *Mycobacterium tuberculosis* culture positive at Hunan Chest Hospital, the biggest TB reference hospital in South-central China, from January 2013 to December 2021. The multiple demographic, clinical and drug susceptibility data of patients were collected from the hospital’s electronic patient records. Descriptive statistical methods, Chi-square test and logistic regression analysis were employed as statistical methods.

**Results:**

Of the 577 LNTB cases, 373 (64.64%) were males, 352 (61.01%) were farmers; majority (161, 33.10%) aged at 20–29 years old; 147 (25.48%) had simple LNTB, 350 (60.66%) had LNTB combined with pulmonary TB (PTB) (defined as LNTB-PTB), and 80 (13.86%) had LNTB combined with other extrapulmonary TB (EPTB) (defined as LNTB-EPTB). A total of 345 (59.79%, 345/577) LNTB patients had cervical node infection, and the simple LNTB patients (81.63%, 120/147) had higher proportion of this infection than LNTB-PTB (51.71%, 181/350) and LNTB-EPTB (55.00%, 44/80) (both *p* values <0.017), respectively. LNTB-EPTB was more inclined to have abdominal tuberculous LNs (20%, 16/80) and at least four tuberculous lesions (22.50%, 18/80) than simple LNTB and LNTB-PTB. Seventy-seven (13.34%) and 119 (20.62%) were resistant to rifampicin (RIF) and isoniazid (INH), respectively; 72 (12.48%) were multi-drug resistant (MDR), and a total of 150 (26.00%) were DR (resistant to at least one of RIF, INH, ethambutol and streptomycin). LNTB patients aged 30–34 and 50–54 years old (compared to those aged <30 years) were independent predictors of RIF resistance (RR) (ORs were 3.47 and 2.83, respectively; 95% CIs were 1.64–7.35 and 1.08–7.46, respectively).

**Conclusion:**

Our study disclosed the epidemiological and DR characteristics of LNTB in Hunan Province, China. High LNTB prevalence was found in younger people while high RR LNTB prevalence was found in older ones, suggesting that we should conduct further studies to clarify the occurrence of RR in LNTB, meanwhile, strengthen the diagnoses and treatments of LNTB to prevent the emergence of RR.

## 1 Introduction

Tuberculosis (TB) is a widespread infectious disease caused by *Mycobacterium tuberculosis* complex (MTBC). In 2022, the number of TB new cases worldwide is as great as 10.6 million and approximately 1.3 million people died from TB (including 167, 000 deaths living with HIV) (1). In China, one of the 30 high TB burden countries in the world (1), TB remains a major public health problem despite a series of control measures since 1990. In 2014, the World Health Organization proposed the End TB Strategy expecting to reduce the incidence of TB by 90% and mortality by 95% by 2035 compared to 2015 (2). Pulmonary TB (PTB) is the most prevalent form that threatens people’s health, however, the disease burden caused by the extrapulmonary TB (EPTB) could not be neglected (2, 3).

EPTB was reported to account for 8–24% of all TB, and even higher percentages in HIV-infected persons (4–6). Among the subtypes of EPTB, lymph node TB (LNTB) was reported as one of the most common forms (7). LNTB poses a great threat to people’s health, it always occurs in the neck, groin, axilla, mesentery, etc. People aged ≤15 years old were more inclined to have LNTB (7, 8). Unlike PTB, only 4–11% of LNTB patients (except those concurrent with other tuberculous infection sites) have systemic symptoms including fever, weight loss, night sweats and mild fatigue (9). The LNs usually present as hard, non-pressure pains with no cutaneous overlay of erythema at the early stage of the MTBC infection and progressive sclerosis and necrosis or subsequent suppuration and spontaneous drainage (10), while only a few LNTB patients escape. Furthermore, the recurrence rate of LNTB after treatment is about 3–20% regardless of the treatment modality, and the pathogenesis of LNTB remains incompletely understood (11). In addition to bacterial factors, the LNTB epidemiology is influenced by host factors such as age, gender, nutrition, genetics, family exposure history and immune competence, which result in different clinical and morphological presentations (12).

According to recent nationwide or multi-center clinical epidemiological investigations of EPTB in China (13, 14), the top five most common EPTB were tuberculous pleurisy, bronchial TB, LNTB, tuberculous meningitis and tuberculous peritonitis, of which, LNTB accounted for 7.24–9.97% and usually happened in LNs of the neck, mediastinum, axilla and lung hilum. In contrast, a study from Australia (15) showed that LNTB accounted for 33.33% of all TB patients, suggesting that the distribution of LNTB varies globally.

Hunan province, located in the South-central of China, is the seventh-population province with a total population of 72.463 million (16) and is a large agricultural province with majority of the population concentrated in rural areas in China. Hunan is also one of the highest TB burden provinces in China and has an estimated TB incidence of 94/100,000, which is higher than the national average (66/100,000) (10). The prevalence of LNTB in Hunan province was only partly estimated in a national study conducted by Li et al. (7), and the results showed that LNTB took a considerable proportion in Hunan province. The drug resistance (DR) profiles of LNTB had never been reported in Hunan province before. However, the DR of TB in Hunan province cannot be ignored, a previous study showed that among the sputum smear-positive TB patients in Hunan province, the DR rate was 37.62% (728/1935) and multi-drug resistance (MDR) rate were 24.13% (17). Therefore, this study aimed to reveal the clinical epidemiology and DR characteristics of LNTB in Hunan province using traditional epidemiological methods, which will contribute to future etiological and pathological studies as well as bring new insights and ideas for the prevention, diagnosis, treatment and management of LNTB.

## 2 Materials and methods

### 2.1 Ethics statement

This study was established according to the ethical guidelines of the Helsinki Declaration and approved by the ethics committee of Hunan Chest Hospital (LS2021051807). This study used data collected from patient records while maintaining patient anonymity. Because this study presented no more than minimal risk of harm to patient subjects, the institutional review board approved a waiver of patient informed consent.

### 2.2 Source of patients and *Mycobacterium tuberculosis* isolates

In this study, we, respectively, enrolled LNTB patients and their isolates from Hunan Chest Hospital during the period 2013–2021. Hunan Chest Hospital is a specialized hospital for TB treatment in Hunan province and one of the sentinel sites for TB surveillance, also delivers treatments for cardiac diseases and thoracic tumors and has a total of 1,600 beds. This hospital provides tertiary care for TB patients from Hunan Province and parts of surrounding regions and accepts severe TB case referrals for patients originating from other regions of China. In addition, the hospital, under the oversight of the hospital authority, ensures compliance with strict protocols for patient care.

The inclusion criteria of LNTB: tuberculous LNs in the chest (including mediastinum and lung hilum), axilla, head, neck, abdomen, or other sites of the body, were all classified as LNTB and included, in other words, all patients with tuberculous LNs with or without other organs’ or tissues’ tuberculous lesions were all included; the patients with repeated hospitalization histories within 1 year were treated as one case. For the convenience of analysis, we classified the LNTB into three groups: (1) simple LNTB group, had TB lesions only in LNs except in mediastinum and lung hilum LNs; (2) LNTB-EPTB group, had LNTB and other types of EPTB, but had no PTB; (3) LNTB-PTB group, had LNTB and PTB regardless of combining other types of EPTB, PTB means that patients had tuberculous lesions in lung, respiratory tract, bronchi, pleura, mediastinum or lung hilum (18), the patients had only tuberculous mediastinum or lung hilum LNs were classified as LNTB-PTB. The exclusion criteria: (1) the patients from other provinces; (2) the patients with incomplete case data; (3) the patients with negative bacterial cultures.

We collected demographic and clinical data, including gender, age, occupation, place of residence, diagnosis results and drug susceptibility profiles from the electronic patient records. We only included drug susceptibility data of streptomycin (SM), isoniazid (INH), rifampicin (RIF) and ethambutol (EMB), while did not include that of pyrazinamide (PZA) for that less than half of the isolates have been tested for PZA susceptibility. All sensitive patients’ information was removed before analysis. For the patients who had multiple isolates, we only included the first isolates for the analysis of DR characteristics, we also analyzed the acquired DR in patients that have multiple isolates acquired at intervals of more than 1 month.

### 2.3 Culture and drug susceptibility testing

Laboratory examination was performed in the clinical laboratory of Hunan Chest Hospital, Changsha, China, a province-level reference laboratory specializing in MTBC detection and equipped with a full set of mycobacterium-detection facilities. Clinical specimens, including sputum, ascites, lavage fluid, pus, feces, and so on, were collected from TB patients and used to isolate MTBC with Lowenstein-Jensen (L-J) medium under 37°C in a biochemical incubator or 7H9 broth medium with Bactec MGIT 960 mycobacterial detection instrument (Becton Dickinson Microbiology System, United States). All isolates were identified as MTBC strains by performing a growth test on 0.5mg/mL p-Nitrobenzoic Acid containing L-J medium (Baso, Zhuhai, China) or by an MBP64 antigen detection kit (Genesis, Hangzhou, China).

Drug susceptibility testing (DST) was performed using a Bactec MGIT SIRE kit (Becton Dickinson) in a Bactec MGIT 960 instrument following the manufacturer’s instructions. All steps were performed by well-trained technicians in a biosafety cabinet in accordance with the relevant guidelines. The reference strain H37Rv was used for quality control once a month or for each new batch of susceptibility kit. The critical concentrations in the MGIT 960 tubes were 1.0 μg/mL for SM, 0.1 μg/mL for INH, 1.0 μg/mL for RIF, 5.0 μg/mL for EMB (12).

### 2.4 Statistical analysis

Statistical analysis of data was done with SPSS (version 21.0, SPSS Inc., Chicago, IL, United States). Descriptive statistics methods (mean, standard deviation, median, frequency, ratio, minimum and maximum) were used to present the population distribution characteristics. Association between categorical variables was evaluated using Chi-square test or Mann-Whittey U test. Odds ratio (OR) with a 95% confidence interval (CI) was determined by logistic regression models. Multivariable logistic regression models were used to evaluate demographic or clinical factors associated with each evidence-based group as applicable. The statistical significance was established at *p* ≤ 0.05. Mapping of the spatiotemporal features was conducted by ArcGIS software (version 10.7, ESRI, Redlands, CA, United States).

## 3 Results

### 3.1 The overall epidemiology of lymph node tuberculosis

**Figure 1:**
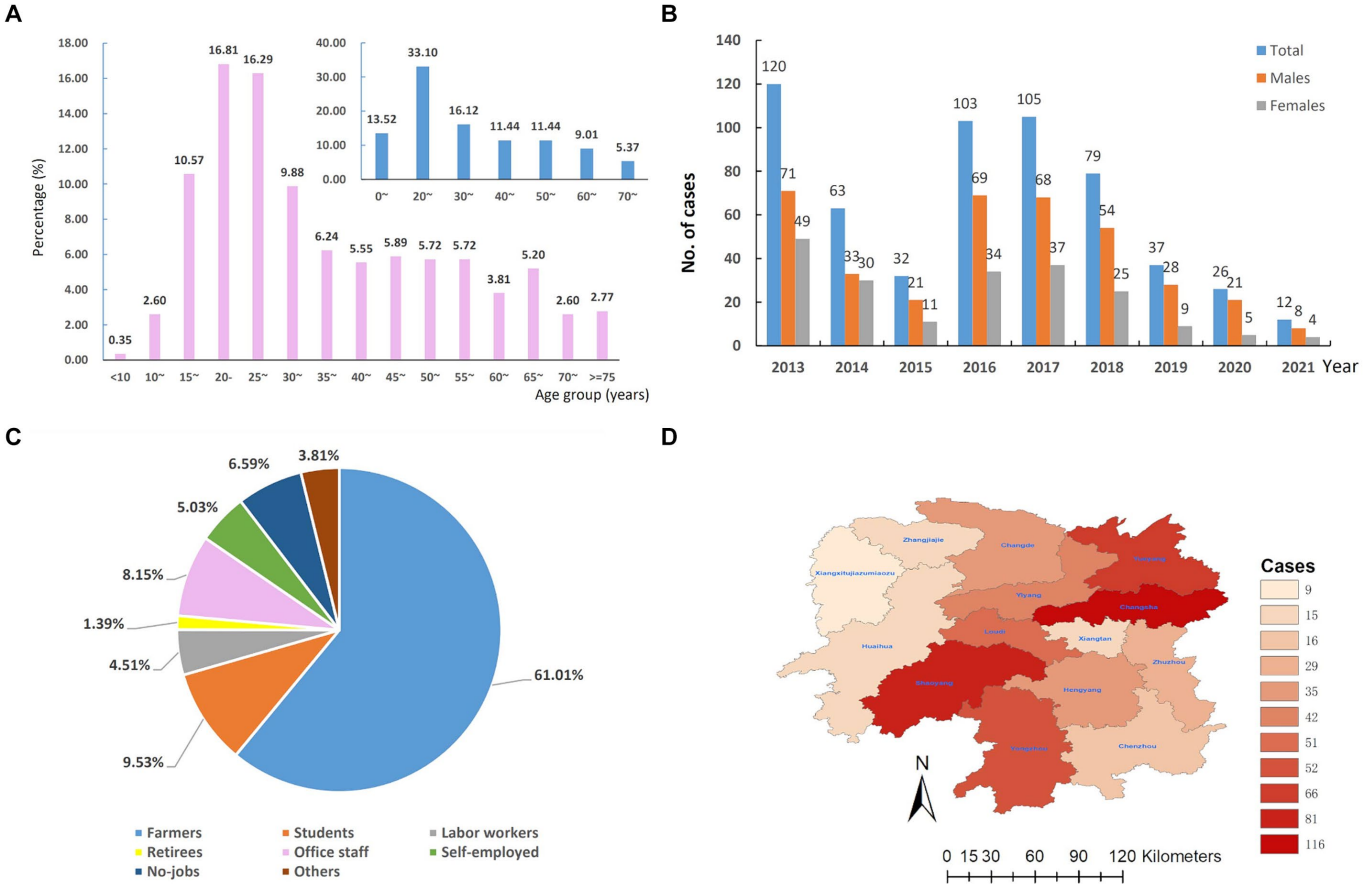
The overall epidemiology of lymph node tuberculosis in Hunan province, China from 2013–2021. **(A)** Age distribution. **(B)** Temporal distribution. **(C)** Occupational distribution. **(D)** Regional distribution.

We totally included 577 patients with LNTB concurrent with or without tuberculous lesions in other organs or tissues of the body in Hunan Chest Hospital from 2013 to 2021, accounting for 43.58% (577/1324) EPTB. These patients were comprised of 373 (64.64%) males and 204 (35.36%) females (male-to-female ratio, 1.83:1), aged from 3 to 83 years old with an average age of 36.93 ± 17.57 years and a median age of 31 (25–75%: 23–50) years. We further analyzed the age-specific LNTB incidence using five-year interval as a group, and found that the 20–24 years age group showed the highest prevalence (97/577, 16.81%) followed by the 25–29 years age group (94/577, 16.29%), and both males and females showed similar age-specific incidence trends (Figure 1A). In total, patients aged 20–29 years old accounted for 33.10% (161/577) of LNTB patients, which was highest among the groups that used 10 years old as a group (Figure 1A).

As shown in Figure 1B, the number of LNTB cases decreased by 73.33% from 2013 to 2015, from 120 cases to 32 cases, however, in 2016 and 2017, the cases of LNTB increased to 103 and 105, respectively, and then dropped during 2019 to 2021. We also analyzed the gender-specific temporal trend of LNTB and found that the number of male patients was consistently more than that of females during the period 2013–2021.

In terms of occupations, the majority of LNTB cases were farmers (61.01%, 352/577), followed by students (9.53%, 55/577), office staff (8.15%, 47/577) and no-jobs (6.59%, 38/577), the self-employed (5.03, 29/577), labor workers (4.51%, 26/577), retirees (1.39%, 8/577) and others (3.81%, 22/577), see Figure 1C.

In terms of regional distribution, 116 (20.10%) were from Changsha City, 81 (14.04%) from Shaoyang, 66 (11.44%) from Yueyang, 52 (9.01%) from Yongzhou, 51 (8.84%) from Loudi, and 211 (36.57%) from other nine cities (autonomous prefecture) of Hunan province. The results are shown in Figure 1D.

### 3.2 The epidemiological characteristics of the subtypes of lymph node tuberculosis

As shown in Table 1, among the 577 patients with LNTB, 147 (25.48%) had simple LNTB, 350 (60.66%) had LNTB-PTB, and 80 (13.86%) had LNTB-EPTB. Cervical LNTB was the most common infection, a total of 345 (59.79%, 345/577) LNTB patients had this infection.

**Table 1:** The epidemiological characteristics of the subtypes of lymph node tuberculosis.

Factors	Simple LNTB (*n*, %)	LNTB-PTB (*n*, %)	LNTB-EPTB (*n*, %)	*χ^2^*	*P*
Gender				4.37	0.112
Males (*n* = 373)	92 (62.60)	221 (63.10)	60 (75.00)		
Females (*n* = 204)	55 (37.40)	129 (36.90)	20 (25.00)		
Age groups (years)				45.31	<0.001
<20 (*n* = 78)	27 (18.40)	42 (12.00)	9 (11.30)		
20 ~ (*n* = 191)	71 (48.30)^a^	96 (27.40)^b^	24 (30.00)^b^		
30 ~ (*n* = 93)	20 (13.60)	57 (16.30)	16 (20.00)		
40 ~ (*n* = 66)	8 (5.40)^a^	52 (14.90)^b^	6 (7.50)^a,b^		
50 ~ (*n* = 66)	12 (8.20)	45 (12.90)	9 (11.30)		
60 ~ (*n* = 52)	4 (2.70)^a^	35 (10.00)^b^	13 (16.30)^b^		
70 ~ (*n* = 31)	5 (3.40)	23 (6.60)	3 (3.80)		
Region				7.10	0.526
East of Hunan (*n* = 160)	39 (26.50)	104 (29.70)	17 (21.30)		
South of Hunan (*n* = 103)	28 (19.00)	65 (18.60)	10 (12.50)		
West of Hunan (*n* = 39)	11 (7.50)	23 (6.60)	5 (6.30)		
North of Hunan (*n* = 101)	23 (15.60)	61 (17.40)	17 (21.30)		
Center of Hunan (*n* = 174)	46 (31.30)	97 (27.70)	31 (38.80)		
Occupations				13.56	0.001
Farmers (*n* = 352)	71 (48.30)^a^	227 (64.86)^b^	54 (67.50)^b^		
Other groups (*n* = 170)	76 (51.70)^a^	123 (35.14)^b^	26 (22.50)^b^		
Location of tuberculous LNs				108.61	<0.001
Neck (*n* = 345)	120 (81.63)^a^	181 (51.71)^b^	44 (55.00)^b^		
Chest (*n* = 67)	0 (0.00)^a^	67 (19.14)^b^	0 (0.00)^a^		
Abdomen (*n* = 42)	5 (3.40)^a^	21 (6.00)^a^	16 (20.00)^b^		
Multiple LNs (*n* = 70)^*^	1 (0.68)^a^	57 (16.29)^b^	12 (15.00)^b^		
Armpit (*n* = 12)^#^	8 (5.44)	2 (0.57)	2 (2.50)		
Unclear (*n* = 41)^#^	13 (8.84)^a^	22 (6.29)^b^	6 (7.50)^a, b^		
Number of tuberculous lesions^$^				556.17	<0.001
1 (*n* = 146)	143 (97.28)^a^	3 (0.86)^b^	0 (0.00)^b^		
2 (*n* = 289)	3 (2.04)^a^	241 (68.86)^b^	45 (56.25)^b^		
3 (*n* = 92)	1 (0.68)^a^	74 (21.14)^b^	17 (21.25)^b^		
≥4 (*n* = 50)	0 (0.00)^a^	32 (9.14)^b^	18 (22.50)^c^		

LNs, lymph nodes; LNTB, lymph node tuberculosis; LNTB-PTB means patients had LNTB and pulmonary TB (PTB) regardless of combining other types of extra pulmonary TB (EPTB); LNTB-EPTB means patients had LNTB and other types of EPTB, but has no PTB. The superscript letters (number^a/b/c^) were used to show the results of one by one comparison, of one specific factor, both groups with the same letter mean that no significance was found, in contrast, there was statistical significance between groups. ^*^Multiple LNs mean that the patients have tuberculous lymph nodes in multiple organs, tissues or systems. ^#^For the convenience of performing RхC Chi-square test, these two groups were combined. ^$^One tuberculous site was seen as a lesion in the present study.

#### 3.2.1 Distributions of gender, age, occupation and region among the subtypes of lymph node tuberculosis

According to the locations of the 14 cities (autonomous prefecture) in Hunan province, we divided Hunan into five areas: east (Changsha, Xiangtan and Zhuzhou), south (Hengyang, Yongzhou and Chenzhou), west (Zhangjiajie, Xiangxi and Huaihua), north (Yueyang and Changde), and center (Loudi, Yiyang and Shaoyang) and compared the region source and gender difference among the three subtypes of LNTB, however, there was no statistical significance (Table 1).

The frequency of simple LNTB was higher than that of both LNTB-PTB and LNTB-EPTB in the age group of 20–29 years, while lower than that of LNTB-PTB in the 40–49 years age group, and lower than that of both LNTB-PTB and LNTB-EPTB in the 60–69 years age group (Table 1).

For the convenience of analysis, we reclassified the occupations as follows: farmers and other groups. Both LNTB-PTB and LNTB-EPTB were more frequent as farmers than simple LNTB (Table 1).

#### 3.2.2 Differences of locations of tuberculous lymph nodes and the number of tuberculous lesions among the subtypes of lymph node tuberculosis

The tuberculous LN locations differed significantly among simple LNTB, LNTB-PTB and LNTB-EPTB (*χ^2^* = 108.61, *p* < 0.001). Results from one by one comparison showed that simple LNTB had higher proportions of tuberculous LNs in the neck than both LNTB-PTB and LNTB-EPTB (81.63% Vs. 51.71 and 55.00%), while LNTB-EPTB was found with more abdominal tuberculous LNs than both simple LNTB and LNTB-PTB (20.00% Vs. 3.40 and 6.00%), and LNTB-PTB and LNTB-EPTB showed similar rates in having multiple LNs (16.29% Vs. 15.00%) (Table 1).

We also found that the tuberculous lesion numbers among the three subtypes of LNTB were significantly different (*χ^2^ =* 556.17, *p* < 0.001). Nearly all simple LNTB patients (97.28%, 143/147) had only one tuberculous lesion, 68.86% (241/350) of the LNTB-PTB and 56.25% (45/80) of the LNTB-EPTB patients had two lesions, LNTB-PTB and LNTB-EPTB patients showed similar proportion in having three lesions (21.14 and 21.25%), whilst 9.14% LNTB-PTB and 22.50% LNTB-EPTB patients had at least 4 lesions (Table 1).

### 3.3 Drug resistance profiles of lymph node tuberculosis

#### 3.3.1 The summary of drug resistance

We totally enrolled 577 patients with LNTB concurrent with or without TB lesions in the other organs or tissues and collected their 793 MTBC isolates. Among the first isolates of the 577 LNTB patients, 427 (74.00%) were susceptible to all four tested drugs (pan-s), 150 (26.00%) were total-DR (means that the isolates were resistant to at least one of four drugs [INH, EMB, RIF and SM]); 54 (9.36%) were Mono-drug resistant (Mono-DR, resistant to only one drug), 72 (12.48%) MDR (resistant to at least INH and RIF), and 24 (4.16%) Poly-DR (resistant to more than one drug but not MDR); 119 (20.62%) were resistant to INH, 106 (18.37%) to SM, 77 (13.34%) to RIF and 35 (6.07%) to EMB.

**Figure 2:**
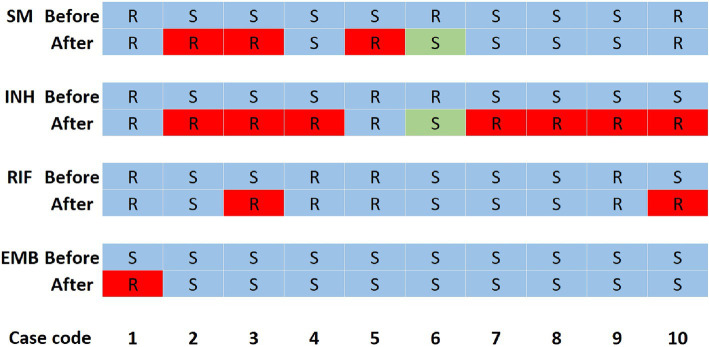
Changes in drug susceptibility results of 10 patients. INH, isoniazid; EMB, ethambutol; SM, streptomycin; RIF, rifampicin; R, resistance; S, susceptibility. R with a red background means the isolates changed from susceptibility to resistance, while S with a green background means the isolates changed from resistance to susceptibility. Before means that this row shows the drug susceptibility patterns of the isolate acquired at least 2 months before the second isolate was acquired. After means that this row shows the drug susceptibility patterns of the isolate acquired at least 2 months after the first isolate was acquired. Each column represents the drug susceptibilities of one patient.

There were 155 individuals with multiple isolates from samples from the same or different body sites, of whom, 113 patients acquired their multiple isolates during the same hospitalization periods (within 14 days) and each individual’s isolates kept consistent drug susceptibility patterns, which indirectly indicated the accuracy of the DST in this hospital. Of the remaining 42 patients who acquired their multiple isolates, respectively, before and after at least 2 months of anti-TB treatments, 10 had inconsistent drug susceptibilities (Figure 2). There were three, seven, two and one case, respectively, acquired resistance to SM, INH, RIF and EMB. It is noteworthy that one case transferred from INH and SM resistance to susceptibility.

#### 3.3.2 Temporal trend of drug resistance of lymph node tuberculosis

**Figure 3:**
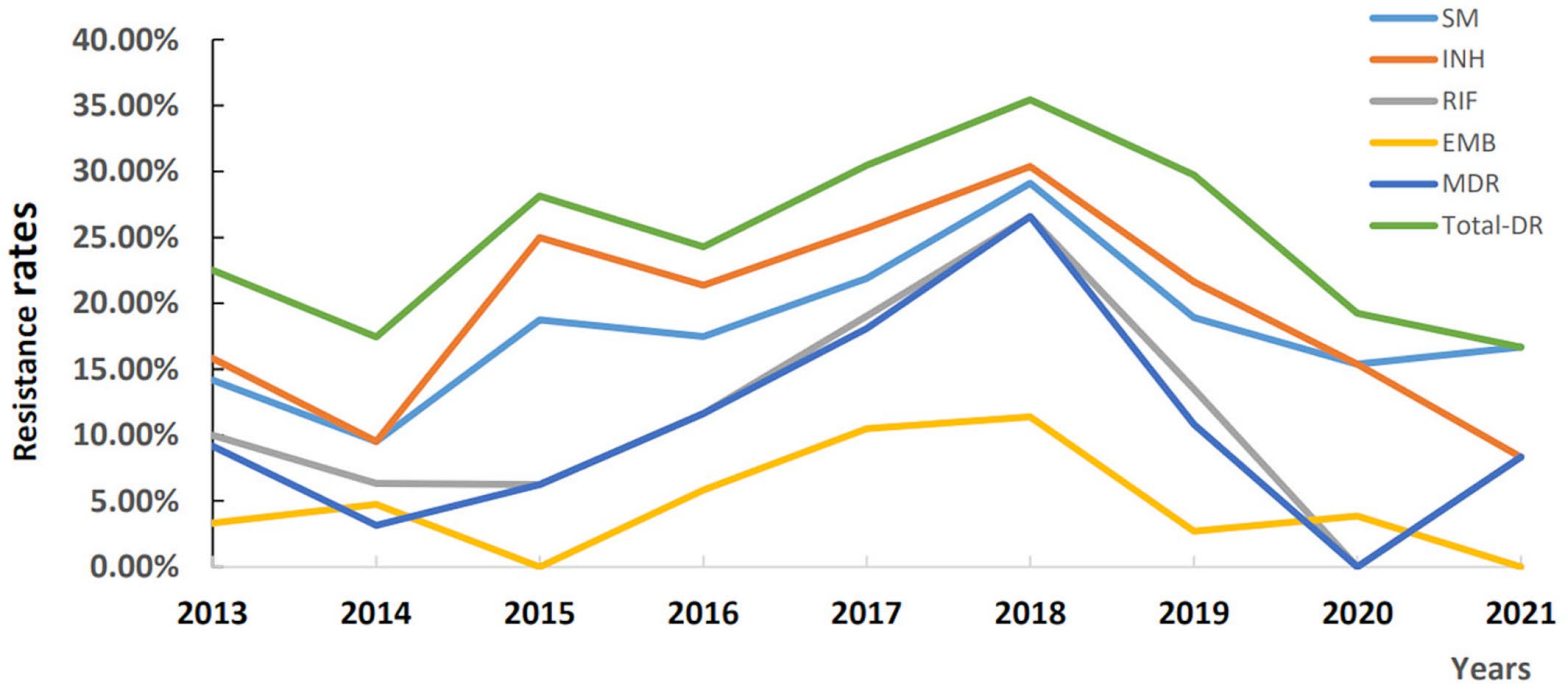
Temporal trends of drug resistance profiles of lymph node tuberculosis in Hunan Province, China from 2013–2021. INH, isoniazid resistance; EMB, ethambutol resistance; SM, streptomycin resistance; RIF, rifampicin resistance; MDR, multi-drug resistance; Total-DR, total drug resistance, means that the isolates were resistant to at least one of the four drugs (INH, EMB, RIF and SM).

The temporal trends of the resistance rates during 2013–2021 were as follows (Figure 3): the trends of total-DR, INH and SM resistance rates were similar, while that of RIF resistance and MDR rates were similar from 2013 to 2021, and all peaked in 2018 and then decreased. It was worth to note the RR and MDR rates increased significantly from 2015 to 2018 and then decreased from 2018 to 2020. EMB nearly showed the lowest resistance among all resistance types from 2013 to 2021, and the EMB resistance rate decreased from 4.76% in 2014 to 0% in 2015, while the other resistance rates began increasing till 2018.

#### 3.3.3 Drug resistance differences among subtypes of lymph node tuberculosis

The RIF and EMB resistance, and MDR rates of LNTB-PTB were all significantly higher than that of simple LNTB (RIF, 16.29% Vs. 6.80%; EMB, 8.00% Vs. 1.36%; MDR, 15.71% Vs. 4.76%), whilst was not significantly different from those of LNTB-EPTB (Supplementary Table S1). We also compared the differences of the six types of DR among LNTB with different LNTB positions, however, no statistical significance was found (The data was not shown).

#### 3.3.4 Drug resistance differences among lymph node tuberculosis with different numbers of tuberculous lesions

The LNTB patients with two tuberculous lesions had higher RIF resistance and MDR rates than that with one (RIF, 16.96% Vs. 6.85%; MDR, 16.61% Vs. 6.79%); whilst those patients with three tuberculous lesions had higher EMB resistance rate than that with one (9.78% Vs. 1.37%) (Supplementary Table S1).

#### 3.3.5 Age-specific drug resistance of lymph node tuberculosis

**Figure 4:**
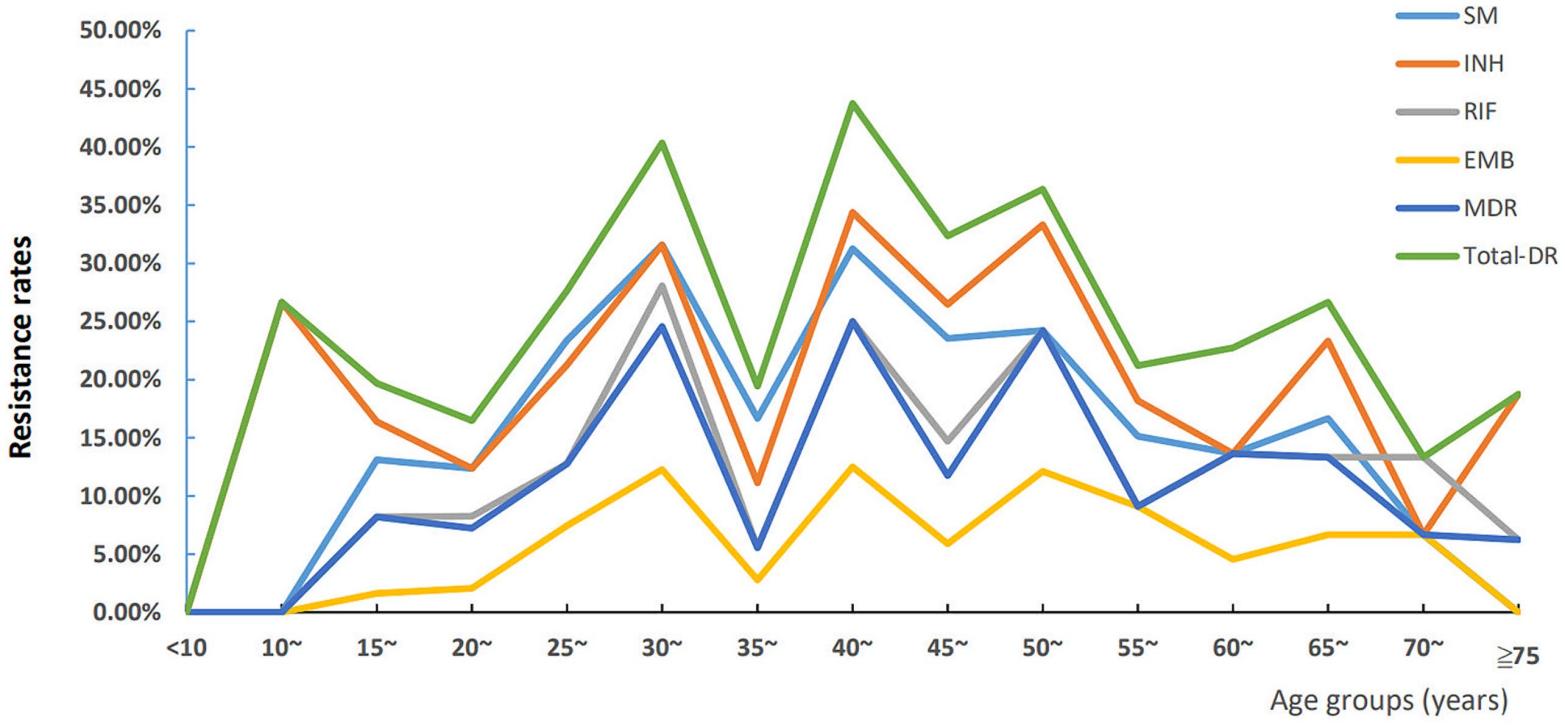
Trends of age-specific drug resistance of lymph node tuberculosis in Hunan Province, China from 2013–2021. INH, isoniazid resistance; EMB, ethambutol resistance; SM, streptomycin resistance; RIF, rifampicin resistance; MDR, multi-drug resistance; Total-DR, total drug resistance, means that the isolates were resistant to at least one of four drugs (INH, EMB, RIF and SM).

We first used five-year interval as a group, the trend of age-specific DR of LNTB was shown in Figure 4, and we found an interesting phenomenon that the six types of resistance rates changed in wavy patterns among the ages of 30 and 55. For statistical analysis, we classified the age groups as follows: <30, 30~, 35~, 40~, 45~, 50 ~ and ≥ 55 years old, and found that each of the six resistance types showed different distribution characteristics of age (Supplementary Table S1). Results from one by one comparison showed that the 30–34 year age group had higher SM and RIF resistance and MDR rates than the <30 year age group (Supplementary Table S1).

#### 3.3.6 Drug resistance differences of regions, genders and occupations

The analysis on the differences of six resistance rates among the LNTB patients from the five areas of Hunan province showed that the rates of SM, INH and RIP resistance, and MDR from the west were, respectively, higher than those from the east and the center, whilst the rates of EMB resistance and total-DR from the west were, respectively, higher than those from the east, and the EMB resistance rate from the west was also higher than that from the north (Supplementary Table S1).

None of resistance rates showed difference between the males and females. The details are shown in Supplementary Table S1.

In terms of occupations, the self-employed showed highest rates of SM (27.59%, 8/29), INH (37.93%, 11/29) and RIF resistance (24.14%, 7/29), and MDR (24.14%, 7/29) and total-DR (37.93%, 11/29), labor workers had highest EMB resistance rate (15.38%, 4/26), however, only the distribution of MDR showed statistically significant difference (*χ^2^* = 9.05, *p* = 0.023) though no statistically significant differences were obtained in pairwise comparisons (Supplementary Table S1).

#### 3.3.7 Factors associated with rifampicin resistance of lymph node tuberculosis by multivariable logistic regression

The RR posed a great threat to the control of TB, we only explored its associated factors. We first included the factors that showed *p* values close to 0.05 (one factor with 0.053) in the univariate analysis into multivariable logistic regression analysis. As shown in Table 2, compared to the LNTB patients aged <30 years old group, patients aged 30–34 and 50–54 years old were positively correlated with RR (ORs were 3.47 and 2.83 respectively; 95% CI were 1.64–7.35 and 1.08–7.46, respectively); compared to the patients from the east of Hunan (Changsha, Xiangtan, Zhuzhou), those from the west of Hunan province were positively correlated with RR (OR = 3.82, 95% CI: 1.49–9.78).

**Table 2:** Factors associated with rifampicin resistance by multivariate logistic regression analysis.

Characteristics	RR LNTB (*n*, %)		*P*	OR (95% CI)
	Yes	No		
Age groups (year)				
<30 (*n* = 269)	25 (9.29)	244 (90.71)	Reference	
30 ~ (*n* = 57)	16 (28.07)	41 (71.93)	**0.001**	**3.47 (1.64–7.35)**
35 ~ (*n* = 36)	2 (5.56)	34 (94.44)	0.315	0.46 (0.10–2.09)
40 ~ (*n* = 32)	8 (25.00)	24 (75.00)	0.081	2.39 (0.90–6.37)
45 ~ (*n* = 34)	5 (14.71)	29 (85.29)	0.604	1.34 (0.45–3.99)
50 ~ (*n* = 33)	8 (24.24)	25 (75.76)	**0.035**	**2.83 (1.08–7.46)**
≧55 (*n* = 116)	13 (11.21)	103 (88.79)	0.775	1.12 (0.51–2.47)
Careers				
Farmers (*n* = 352)	48 (13.64)	304 (86.36)	Reference	
Labor workers (*n* = 26)	6 (23.08)	20 (76.92)	0.190	2.02 (0.71–5.77)
Self-employed (*n* = 29)	7 (24.14)	22 (75.86)	0.244	1.83 (0.66–5.09)
Other groups (*n* = 170)	16 (9.41)	154 (90.59)	0.603	0.84 (0.43–1.64)
LNTB subtypes				
Simple LNTB (*n* = 147)	10 (6.80)	137 (93.20)	0.679	0.57 (0.04–8.17)
LNTB-PTB (*n* = 358)	57 (16.29)	293 (83.71)	0.368	1.43 (0.66–3.12)
LNTB-EPTB (*n* = 80)	10 (12.50)	70 (87.50)	Reference	
No. of tuberculous lesions				
1 (*n* = 146)	10 (6.85)	136 (93.15)	Reference	
2 (*n* = 289)	49 (16.96)	240 (83.04)	0.905	1.17 (0.09–14.78)
3 (*n* = 92)	10 (10.87)	82 (89.13)	0.775	0.68 (0.05–9.44)
≥4 (*n* = 50)	8 (16.00)	42 (84.00)	0.839	1.32 (0.09–19.01)
Region				
East of Hunan (Changsha, Xiangtan, Zhuzhou) (*n* = 160)	16 (10.00)	144 (90.00)	Reference	
South of Hunan (Hengyang, Yongzhou, Chenzhou) (*n* = 103)	19 (18.45)	84 (81.55)	0.055	2.09 (0.98–4.46)
West of Hunan (Zhangjiajie, xiangxi, Huaihua) (*n* = 39)	11 (28.21)	28 (71.79)	**0.005**	**3.82 (1.49–9.78)**
North of Hunan (Yueyang, Changde) (*n* = 101)	13 (12.87)	88 (87.13)	0.411	1.41 (0.62–3.20)
Center of Hunan (Loudi, Yiyang, Shaoyang) (*n* = 174)	18 (10.34)	156 (89.66)	0.741	1.14 (0.54–2.40)

LNTB, lymph node tuberculosis; LNTB-PTB means patients had LNTB and pulmonary TB (PTB) regardless of combining other types of extra pulmonary TB (EPTB); LNTB-EPTB means patients had LNTB and other types of EPTB, but has no PTB. RR, rifampicin resistance. Numbers in bold mean that these factors emerged as statistically significant observations.

## 4 Discussion

LNTB is the most common among all types of EPTB and is often concurrent with PTB. Studies on the epidemiology of LNTB will help to control the epidemic of TB. Our present study focused on the clinical epidemiological and drug resistance characteristics of LNTB in Hunan province from 2013 to 2021, the results greatly enriched such knowledge.

The present study found that of the 577 LNTB patients, 64.64% were males and 35.36% were female, the gender ratio (1.83:1) was similar to that in local TB cases (19). Li et al. (7) reported that more females were found in the EPTB patients and Pang et al. (13) reported that the number of female LNTB cases was more than four times that of males, suggesting the gender profile of LNTB may be different from that of other EPTB and there was an areal feature on the LNTB incidence.

A study from Mexico showed that EPTB has a greater impact on people aged 2–15 years (8). A national study on EPTB from China showed that the LNTB’s incidence was the highest at ages 0–15 years and then decreased while age increased (7). However, the present study found the 20–29 years age group had the highest proportion (161/577, 33.10%), the reason for the difference may be due to the sample source difference: the patients in this study were from a provincial reference TB hospital, where the admissions are mainly adults, however, the pediatric patients in this area are more likely to visit children’s hospitals, which resulted in low LNTB prevalence in adolescents and children in the present study. We also found that LNTB patients in some age groups preferred to have one or two of the three subtypes of LNTB, e.g., the 20–29 years age group usually got simple LNTB, whereas the 40–49 years age group were prone to have LNTB-PTB and the 60–69 years age group have either LNTB-PTB or LNTB-EPTB, suggesting that the symptoms of TB become more complicated and may be associated weakened immunity with age increase.

The main occupation of LNTB in the present study was farmers (accounting for 61.01%), which may be attributed to that Hunan province is a large agricultural province, the majority of the population is in rural areas, and farmers with severe LNTB usually go to the provincial TB hospital and seek for treatments. A national survey of DR-TB in China conducted in 2007–2008 found that 65.21% (1,310/2009) of TB patients were farmers (20), which was similar to our study. Another study from Beijing from 2012 to 2019 reported that 32.85% (7,552/22988) of all TB patients and 77.89% (310/399) of tuberculous meningitis patients were farmers (21). These evidences suggested that the burden of TB especially EPTB on farmers in China should be paid more attentions to. It was worth noticing that students were found as the second group in our study, as students spend most of their time together in relatively closed classrooms or dormitories, which would increase the spread of infection and lead to the outbreak of TB (22). Therefore, we should strengthen TB prevention and control in schools.

Analysis of the regional distribution of cases showed that the LNTB cases were mainly from Changsha City, Shaoyang City, and Yueyang City. The reason for this phenomenon may be due to the population density difference among the cities (autonomous prefecture) in Hunan Province. Besides, Hunan Chest Hospital located in Changsha, LNTB patients from the local area and surrounding areas were more preferred to seek treatment in this hospital. The high prevalence of LNTB in Shaoyang and Yueyang suggested that we should pay more attention to the control of LNTB in these areas.

The temporal trend of LNTB showed decrease trends during 2013 to 2015, the explanations may be that: first, Hunan province implemented 10 health measures to benefit the people in 2013, including optimization the New Rural Cooperative Medical Service (NRCMS) compensation policy, expanding the reimbursement scope, and improving the compensation level of hospitalization; second, the government provided free anti-TB treatment for patients with active TB (23). Among 2016 and 2017, the LNTB cases were, respectively, threefold of those in 2015, which may be attributed to the applications of the new molecular diagnosis tools (24), and comprehensive prevention and treatment service model in these years (25). Since 2018, the LNTB cases showed a decreased trend, especially during 2020–2021. The low LNTB prevalence between 2020 and 2021 may be due to a decrease in patient hospitalization as the COVID-19 (26). A significant decrease in the reported incidence of respiratory infectious diseases in the context of the COVID-19 epidemic has been reported in many countries including Japan (27) and China (28).

Hunan is one of the provinces with high burden of DR-TB in China (29). The proportions of DR-TB among all patients and previously treated TB patients in Hunan were 10.5 and 28.8%, respectively (30). In this study, among the LNTB patients, the total-DR accounted for 26.0%, which was close to the rate (28.8%) reported in previously treated patients (30). RIF is one of the key drugs for TB treatment, and the incidence of RR is an important indicator of the severity of local DR-TB. We found that the RR rate in LNTB was 13.34%, about twice higher than the national level (5.71%) in the new TB cases in China (20). We also found that INH owned the highest resistance rate (20.62%) among the four tested anti-TB drugs, higher than that among the new cases (16.0%) while lower than that among the previously treated cases (38.5%) from a national drug surveillance of China (20). Previous studies showed that patients with INH resistance have higher risk of developing PZA resistance and MDR, and are inclined to result in poor treatment outcomes (17, 31, 32). These evidences suggest that DR in LNTB was severe and should be noticed by the clinicians and public health departments in Hunan Province.

In terms of the temporal trends of DR rates during 2013–2021, we found that all resistance rates peaked in 2018, which may be attributed to the promulgation of a TB management policy in Hunan province in 2018 (33): according to the “13th Five-Year Plan” for TB prevention and control in Hunan province, each county and city were required to set up one to two TB designated medical institutions, and all of 14 cities (autonomous prefecture) in Hunan province were required to establish hospitals for DR-TB diagnosis and treatment to convenient DR-TB patients to seek for medical care in local areas. So, as a provincial DR-TB-designated hospital, lots of DR-LNTB patients were transferred here, which led to the peak of all types of resistance rates in 2018.

In the present study, nine out of 42 patients with multiple isolates from their different hospitalization periods acquired INH, RIF, EMB or (and) SM resistance with acquired INH resistance were most common (Figure 2), suggesting the importance of surveillance of drug susceptibilities during treatment in order to adjust the therapy. One patient changed from Poly-DR (INH + SM resistance) to pan-s after treatment, which may be due to the re-infection or restoration of INH and SM resistance, and further genotyping and DST are needed to confirm. Richardson et al. (34) reported that a laboratory-constructed *H37Rv ΔkatG::katG W300G* mutant reverted from an INH-resistant to a susceptible phenotype and from GGG (glycine, G) to TGG (tryptophan, W) in the absence of INH therapy. The EMB resistance reversity was also found in a *orn-embB-aftA M. tuberculosis* mutant (35).

A previous study showed that age older than 60 years was a predictor of DR-TB with univariate analysis (36). In this study, according to the age-specific DR rate analysis, the age group of 30–34 years owned the highest rates of MDR, RIF and SM resistance. For the RR LNTB, after adjusting for potential confounding factors using multivariate logistic regression, we found that patients aged 30–34 and 50–54 years old were independent predictors of RR (ORs were 3.47 and 2.83 respectively; 95% CI were 1.64–7.35 and 1.08–7.46, respectively), while compared to the LNTB patients aged <30 years old group. Lv et al. (37) showed that 30–59 years age group showed the highest DR rate, and Wang et al. (38) showed that the MDR-TB detection rate was more common among individuals under 51 years of age (14.1%) than those over 50 years of age (9.3%), these results were partly confirmed by our study.

In this study, we also found that LNTB patients from the west of Hunan China (compared to those from the east of Hunan) were independent predictors of RR. The samples in the present study were from a provincial TB hospital (Hunan Chest Hospital) in Changsha city (located in the east of Hunan), and the west of Hunan is the farthest area from Changsha city, we speculated one reason was that the LNTB patients with serious, DR and/or prolonged treatment without improvement were transferred to this hospital, resulting the higher resistance rates in this area in the present study.

In the present study, we found that LNTB-EPTB had more abdominal tuberculous LNs and more tuberculous lesions, however, we did not find the LNTB-EPTB and the number of tuberculous lesions were associated with DR (except three lesions were associated with EMB resistance), suggesting that severe LNTB may not be due to DR and larger sample size studies are needed to verify, or may be due to other causes, such as diagnostic and therapeutic delays.

There were two limitations in this study. First, we only included the inpatients from the Hunan Provincial Chest Hospital, and the inpatients with severe and DR-TB were more inclined to hospitalization, which may have resulted in a bias on the epidemiology of LNTB, especially overestimated its drug resistance levels. Second, there was no available information on the categories of new cases or relapse, so we could not analyze the resistance rates of each category and provide more exact clues on the management of resistant LNTB.

In conclusion, LNTB was more inclined to happen in males, adults aged 20–29 years old, farmers, and patients from Changsha, Shaoyang and Yueyang cities; simple LNTB usually happened in the neck, LNTB-EPTB were more inclined to carry abdominal tuberculous LNs and ≥ 4 tuberculous lesions than simple LNTB and LNTB-PTB. Total-DR and RR rates of LNTB were 26.00 and 13.34%, respectively; LNTB patients aged 30–34 and 50–54 years old were independent predictors of RR. High LNTB prevalence was found in younger people while high RR LNTB prevalence was found in older ones, suggesting that we should conduct further studies to clarify the mechanism of occurrence of RR in LNTB, meanwhile, pay more attention to the diagnoses and treatments of LNTB to prevent the emergence of RR.

## Data availability statement

The original contributions presented in the study are included in the article/Supplementary material, further inquiries can be directed to the corresponding authors.

## Ethics statement

The studies involving humans were approved by the ethics committee of Hunan Chest Hospital. The studies were conducted in accordance with the local legislation and institutional requirements. Written informed consent for participation was not required from the participants or the participants' legal guardians/next of kin in accordance with the national legislation and institutional requirements.

## Author contributions

TF: Formal analysis, Methodology, Resources, Writing – original draft, Writing – review & editing. YX: Formal analysis, Methodology, Writing – original draft. WL: Methodology, Resources, Writing – review & editing. BL: Formal analysis, Methodology, Resources, Writing – review & editing. HL: Formal analysis, Methodology, Resources, Supervision, Validation, Writing – review & editing. YW: Formal analysis, Methodology, Writing – review & editing. DX: Formal analysis, Methodology, Validation, Writing – review & editing. ML: Formal analysis, Methodology, Validation, Writing – review & editing. SY: Formal analysis, Methodology, Validation, Writing – review & editing. JL: Formal analysis, Methodology, Validation, Writing – review & editing. YY: Formal analysis, Methodology, Resources, Validation, Writing – review & editing. XZ: Formal analysis, Methodology, Validation, Writing – review & editing. L-lZ: Formal analysis, Methodology, Validation, Writing – review & editing. KW: Formal analysis, Methodology, Resources, Validation, Writing – review & editing. XY: Investigation, Methodology, Resources, Supervision, Validation, Writing – review & editing. YT: Conceptualization, Resources, Supervision, Validation, Writing – review & editing. GL: Conceptualization, Data curation, Formal analysis, Funding acquisition, Methodology, Validation, Writing – original draft, Writing – review & editing.
